# CVAR-Seg: An Automated Signal Segmentation Pipeline for Conduction Velocity and Amplitude Restitution

**DOI:** 10.3389/fphys.2021.673047

**Published:** 2021-05-24

**Authors:** Mark Nothstein, Armin Luik, Amir Jadidi, Jorge Sánchez, Laura A. Unger, Eike M. Wülfers, Olaf Dössel, Gunnar Seemann, Claus Schmitt, Axel Loewe

**Affiliations:** ^1^Institute of Biomedical Engineering (IBT), Karlsruhe Institute of Technology (KIT), Karlsruhe, Germany; ^2^Medizinische Klinik IV, Städtisches Klinikum Karlsruhe, Karlsruhe, Germany; ^3^Klinik für Kardiologie und Angiologie II, University Heart Center Freiburg-Bad Krozingen, Bad Krozingen, Germany; ^4^Faculty of Medicine, University of Freiburg, Freiburg, Germany; ^5^Institute for Experimental Cardiovascular Medicine, University Heart Center Freiburg-Bad Krozingen, Freiburg, Germany

**Keywords:** restitution, atrial tissue characterization, conduction velocity, amplitude, signal segmentation, cardiac electrophysiology, S1S2 stimulation protocol

## Abstract

**Background:**

Rate-varying S1S2 stimulation protocols can be used for restitution studies to characterize atrial substrate, ionic remodeling, and atrial fibrillation risk. Clinical restitution studies with numerous patients create large amounts of these data. Thus, an automated pipeline to evaluate clinically acquired S1S2 stimulation protocol data necessitates consistent, robust, reproducible, and precise evaluation of local activation times, electrogram amplitude, and conduction velocity. Here, we present the CVAR-Seg pipeline, developed focusing on three challenges: (i) No previous knowledge of the stimulation parameters is available, thus, arbitrary protocols are supported. (ii) The pipeline remains robust under different noise conditions. (iii) The pipeline supports segmentation of atrial activities in close temporal proximity to the stimulation artifact, which is challenging due to larger amplitude and slope of the stimulus compared to the atrial activity.

**Methods and Results:**

The S1 basic cycle length was estimated by time interval detection. Stimulation time windows were segmented by detecting synchronous peaks in different channels surpassing an amplitude threshold and identifying time intervals between detected stimuli. Elimination of the stimulation artifact by a matched filter allowed detection of local activation times in temporal proximity. A non-linear signal energy operator was used to segment periods of atrial activity. Geodesic and Euclidean inter electrode distances allowed approximation of conduction velocity. The automatic segmentation performance of the CVAR-Seg pipeline was evaluated on 37 synthetic datasets with decreasing signal-to-noise ratios. Noise was modeled by reconstructing the frequency spectrum of clinical noise. The pipeline retained a median local activation time error below a single sample (1 ms) for signal-to-noise ratios as low as 0 dB representing a high clinical noise level. As a proof of concept, the pipeline was tested on a CARTO case of a paroxysmal atrial fibrillation patient and yielded plausible restitution curves for conduction speed and amplitude.

**Conclusion:**

The proposed openly available CVAR-Seg pipeline promises fast, fully automated, robust, and accurate evaluations of atrial signals even with low signal-to-noise ratios. This is achieved by solving the proximity problem of stimulation and atrial activity to enable standardized evaluation without introducing human bias for large data sets.

## Introduction

Atrial fibrillation (AF) is the most common supraventricular cardiac arrhythmia, with a fourfold increase in prevalence over the last 50 years (Schnabel et al., 2015). Treatment strategies remain suboptimal in terms of efficiency and outcome (Hindricks et al., 2020). Fibrosis, among other effects, refers to an excess deposition of collagen inside the cardiac tissue (Staerk et al., 2017; Lawson et al., 2020) and is suspected to be responsible for maintaining arrhythmias, e.g., due to anchoring of reentrant depolarization waves (Jadidi et al., 2020; Heijman et al., 2021).

Current invasive AF treatment strategies use multi-electrode catheters to record electrograms and characterize the substrate by the peak-to-peak amplitude (often simply distinguishing between “high” and “low” voltage) and conduction velocity (CV). This information helps to guide the ablation procedure. It has been shown that fibrotic tissue correlates with regions of low voltage (Jadidi et al., 2013; Rodríguez-Mañero et al., 2018; Caixal et al., 2020). Beyond pulmonary vein isolation, these low voltage regions are commonly targeted by ablation (Nielsen et al., 2014; Carrizo and Morillo, 2016; Schade et al., 2020). A drawback of the low voltage guided approach is that it ignores the rate-dependent nature, i.e., restitution information, of both amplitude and CV. Restitution is the property that as the diastolic interval of a proceeding beat varies the action potential duration (APD) and CV of the current beat also vary. In general, a decrease of diastolic interval is followed up with a decrease in APD and CV. On a cellular level this is caused by an incomplete recovery of the membrane voltage to the resting potential, which in turn reduces sodium channel availability, thereby reducing sodium influx and upstroke velocity of the following beat. Due to its potential as a predictor for AF and AF recurrence (Narayan et al., 2008; Ramírez and Tinker, 2020), restitution is an active field of research. Furthermore, fibrotic regions are present in paroxysmal as well as persistent AF patients (Verma et al., 2005) hinting at a more complex interconnection. Subtypes of AF might be uncovered by using restitution information.

Both the CV and amplitude restitution curves have the potential to reflect and uncover the underlying mechanisms related to the initiation and perpetuation of atrial and ventricular arrhythmias (Fenton et al., 2002; Xie et al., 2009). Compared to a healthy control group, the APD restitution curves in AF patients are shifted toward lower values and exhibit a less steep slope for basic cycle lengths (BCL) under 400 ms (Franz et al., 1997). Furthermore, Xie et al. (2009) reported that an increase in fibrosis resulted in a shift of the CV restitution curve toward lower CV values. Therefore, the slope and asymptote of the restitution curve are connected to substrate characteristics and could be used for diagnostical purposes, or can be used to monitor drug intervention to adjust the slope beyond a threshold (Franz, 2003).

For restitution studies in clinical practice, rate-varying S1S2 stimulation protocols are predominantly used (Narayan et al., 2008). They minimize the risk of inducing AF that continuous high frequency pacing carries. S1S2 protocols allow measuring the immediate electrophysiological response to a change in pacing coupling interval (Kalb et al., 2004). The S1S2 protocol consists of several pacing trains. Each pacing train contains several stimuli with a BCL, called the S1 stimuli, followed by a single stimulus administered after a reduced coupling interval, called the S2 stimulus. This pacing train is repeated, with the S1 stimuli retaining their BCL and a sequentially decreasing coupling interval between the last S1 stimulus and the S2 stimulus. The pacing protocol ends as soon as the atrial tissue does not depolarize at a certain S2 coupling interval anymore because the S2 stimulus falls inside the effective refractory period (ERP).

Multiple challenges arise for an automatic signal processing pipeline. Different clinical hardware setups lead to a morphological variability of signals. Additionally, all variable input parameters (filter and stimulation setup) that the physician can control have to be considered. Lastly, in clinical practice, different protocol settings are used, and ad-hoc deviations from the initial study protocol can be required.

In an EP study, signals may be fractionated due to fibrosis, measurement artifacts, or noise influences, making detection of local activation time (LAT) of active signal segments challenging. Two common methods are the non-linear energy operator (NLEO) (Schilling et al., 2015) and the wavelet transform (Lenis et al., 2016), however, there exist multiple alternative ways of determining the LAT (Cantwell et al., 2015; Schilling et al., 2015; Corrado et al., 2017). These approaches lack the ability to detect atrial activities lower in amplitude and maximum slope than a nearby stimulation artifact, necessitating an additional step of stimulus removal to make them viable if a stimulation artifact partly overlaps with the atrial activity of interest.

These considerations led to three requirements for our S1S2 segmentation pipeline: (i) the pipeline must not rely on a-priori knowledge of the procedural parameters; (ii) an automated pipeline is needed, which segments and annotates time windows of activities and stimulations; (iii) a clean removal of the stimulus artifact is necessary to uncover the underlying atrial activity for local measurements with stimuli originating in proximity to mapping electrodes. The independence of procedural parameters allows for verification of measurement data and saves time by eliminating potentially bad outlier measurements at an early research stage.

To the best of our knowledge, there is no established best practice for an atrial activity segmentation pipeline of S1S2 measurements to date. Such a pipeline must meet high quality standards regarding accuracy, robustness, and reproducibility. To address this issue, we propose the CVAR-Seg pipeline for the automatic segmentation of intracardiac S1S2 protocol measurements to gain temporal (LATs) and spatial (distances) parameters from patient data as a basis for deducing restitution curves of both amplitude and CV. The pipeline was designed modular, to allow users to exchange the existing modules with alternatives for atrial detection, LAT assignment or CV calculation components. By releasing the software as open source, the pipeline can be freely used and adapted.

The pipeline was tested on synthetic signals with a known ground truth. Noise estimated from a clinical EP case was added to the synthetic signal to test the pipeline robustness for increasing noise levels. Finally, we applied the method in a clinical proof of concept with one patient data acquired with the widely used CARTO system.

## Materials and Methods

**FIGURE 1:**
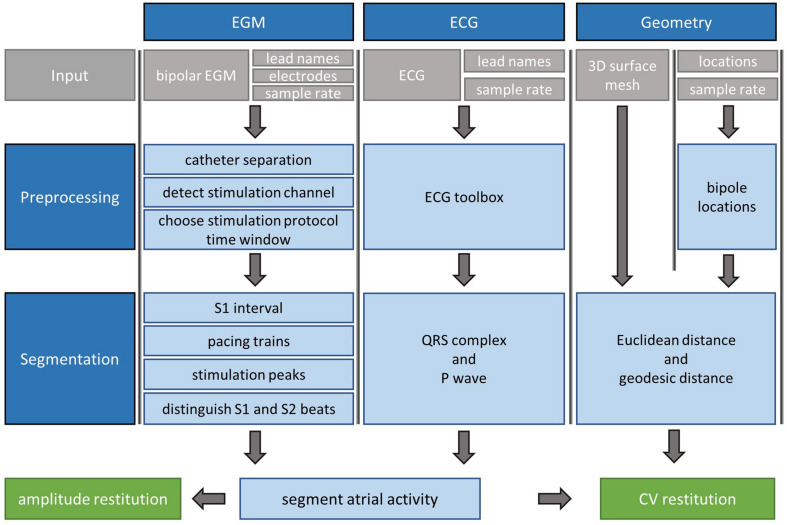
Segmentation pipeline for clinical S1S2 protocol measurements consisting of EGM, ECG, and geometry data. Gray boxes are pipeline input. The pipeline yields segmented atrial activities. In additional steps, CV and amplitude restitution can be calculated.

In this section, an overview of CVAR-Seg (Figure 1) and the basic ideas behind each step are presented. For more detailed information, the interested reader is referred to the Supplementary Material. The pipeline uses electrogram (EGM), electrocardiogram (ECG), and geometry data from clinical S1S2 stimulation routines. From these, the pipeline segments activities and computes amplitude and CV restitution curves.

### Preprocessing of Input Data

#### Electrograms (EGM)

In the first processing step of the segmentation pipeline, different catheters used throughout the experiment are detected, thereby enabling the distinction between stimulating and non-stimulating catheters. Both unipolar and bipolar signal evaluation is supported. Since the bipolar signals are less noisy this is used for all segmentation steps and the final segmentation windows are then evaluated on the unipolar traces. Therefore, from the input the bipolar signals are extracted and used for all further steps. From electrical field theory, we know that the stimulation has the highest amplitude in the stimulating channel, and with increasing spatial distance to the stimulation source, the amplitude decreases. Therefore, neighboring channels should have the second highest amplitudes. This relation is used to detect the stimulating catheter. The channel with the highest signal amplitude and the channel with the second highest amplitude are found and checked if they are spatially neighboring channels. This step adds robustness against high amplitude noise artifacts in non-stimulating channels. The following manual selection of the EGMs evaluation window is turned off for the fully automated processing where the whole input segment is evaluated.

#### Electrocardiogram (ECG)

In the case of the optional use of a corresponding ECG trace, the signal is first up-sampled by linear interpolation to the sampling rate of the EGM signal. ECG segmentation is based on the open source ECGdeli toolbox (Pilia et al., 2020), yielding a fiducial point table comprising 13 crucial time points characterizing the ECG trace. Of those, this pipeline uses the start, peak, and end of the QRS-complex.

### S1S2 Protocol Segmentation

Since the working premise is that all protocol parameters are unknown, the primary goal was to segment the prominent stimulation signals first, to use them as landmarks to obtain atrial activities which are more difficult to segment.

#### Stimulation Detection and S1 Cycle Length Estimation

**FIGURE 2:**
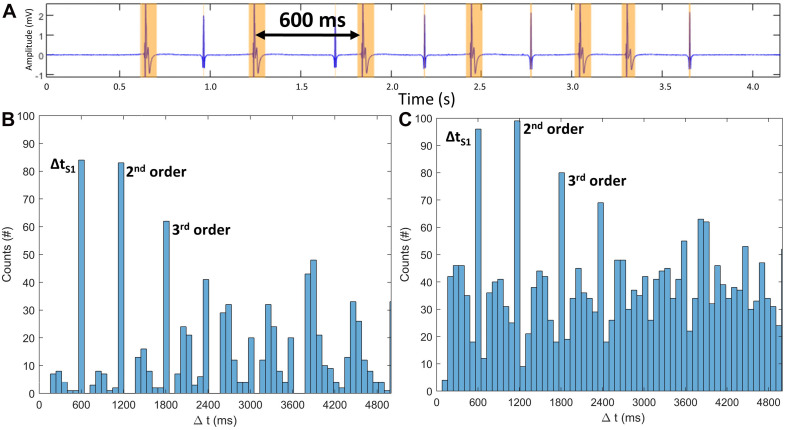
**(A)** Active segment detection (yellow intervals) of a stimulation block segment of a synthetic 80-s-long S1S2 protocol with 20 randomly distributed extra beats – **A** shows 5 extra beats. S1 peaks have a cycle length of 600 ms. **(B)** Histogram of all time intervals between active segments. *x*-axis cut at 5000 ms. Time interval corresponding to S1 cycle length and the harmonics present the highest contribution. **(C)** Fourfold increase of extra beats does not change the three main contributors.

To distinguish stimulation components from physiological signal components, a high pass filter with a cutoff frequency of 450 Hz is applied to exclude most components of biological origin and their low order harmonics (Malmivuo and Plonsey, 2012), retaining stimulation-related peaks in the clinically filtered signal traces. To detect stimulation segments in the so filtered signals, the wavelet method using the bior1.5 mother wavelet following Lenis et al. (2016) is applied. Each channel trace is analyzed, and the temporal start, peak, and end of all active signal segments are annotated. To create exact time points of start and end of active signal segments, a threshold is applied to the resulting wavelet-filtered signal. The threshold was defined as the standard deviation of the amplitudes across all samples in the segment multiplied by a factor *k* that is determined iteratively: starting from a low initial *k* value of 0.01 to include as many active segments as possible (Figure 2A, yellow segments), the wavelet segmentation is performed. A histogram of the temporal distances *Δt* between all peaks is evaluated, and the three most common values are identified (Figure 2B) based on the idea that the S1 cycle length will occur most often in an S1S2 protocol. The second and third highest peaks in the histogram should be integer multiples of the S1 cycle length within a tolerance of 10 samples. This holds true for cases with increased extra beats (Figure 2C). If the integer relationship is confirmed, *k*, the detected segments, and the S1 cycle length are determined. If the second and third highest peaks are not integer-multiples, *k* is successively increased by a *Δk* of 0.01 until a consistent S1 cycle length is identified. Should this condition not be met after a maximum number of iterations a warning is issued. This concludes the estimation of the S1 cycle length by using the S1 time interval detection.

#### Number of Pacing Blocks and Stimuli Estimation

Based on the approach of the previous section, the S1 cycle length is known. However, the time interval between S1S2 pacing trains (i.e., the interval between a S2 stimulus and the following S1 stimulus, Δ*t*_*S*2*S*1_) and the number of S1 stimuli contained within each S1S2 pacing train remain unknown (Supplementary Figure 1 and Supplementary Material section “Number of Pacing Blocks and Stimuli Estimation”). Therefore, the time differences *Δt* between peaks are classified with a k-means clustering algorithm (Lloyd, 1982) using two clusters. This yields one cluster containing the S1 cycle lengths (Δ*t*_*S*1_) and S2 coupling intervals (Δ*t*_*S*2_), respectively, and the second cluster containing the markedly longer S2 to S1 (Δ*t*_*S*2*S*1_) intervals between pacing trains. With this information, different pacing trains can be separated. The mode (most frequent value) of the number of identified successive S1 cycles present is used to identify the number of S1 stimuli within a pacing train. To identify the S2 coupling interval, a time window of interest after the last S1 stimulus is defined with the length of S1 because the S2 interval is normally shorter than S1. The rationale for the statistical k-means approach was that extra beats could impair signal segmentation performed on single segments of the measurement. Also, no knowledge of the time window between pacing trains and the S2 time windows was needed. The only assumption used was that the time between trains is longer than the S1 cycle length.

#### Detection of Stimulus Time Segments

The previous segmentation step detects the time segments of pacing trains. Segmentation of stimulation time segments based solely on time intervals between peaks would be prone to errors in peak detection and noise since an extra beat would disrupt the time interval between neighboring stimuli. For noise-robust stimulus time detection, five conditions (C1–C5) were formulated:

C1: Stimulations are detected in multiple channels of the catheter at the same time.
C2: Stimulations detected by NLEO after high-pass filtering agrees with the detected stimulation time segments by wavelet filtering as detailed in subsection 2.2.1.
C3: In the first principal component computed across all channels, peaks overlap with the detected stimulation time segments and surpass an amplitude threshold of 90% of the maximum first principal component amplitude. This is motivated by the difference of far field effect on the stimulation signal in the spatially varying channels and the fact that the stimulation artifact in the stimulation channel will follow the morphology given by the stimulus generator, which differs in morphology from the far field in the other channels.
C4: The time interval between neighboring stimuli does not deviate from the S1 cycle length by more than 10 samples.
C5: The first principal component of all derivatives of all channel signals overlaps with the detected stimulation time segments and surpasses 90% of the first principal component signal.

The threshold to determine trustworthy stimulation time segments was set such that a stimulation had to be detected in at least half of all channels (C1, C2) and had to pass the derivative (C4) and the amplitude (C3, C5) conditions. Applying that threshold separates the detected time segments into trusted and discarded stimulus time segments. The remaining trusted stimulation time segments are then classified as either S1 or S2 beats using counters or cross-correlation methods. More details on peak detection and code implementation can be found in Supplementary Material section “Detection of Stimulus Time Segments.”

#### Atrial Activity Segmentation

**FIGURE 3:**
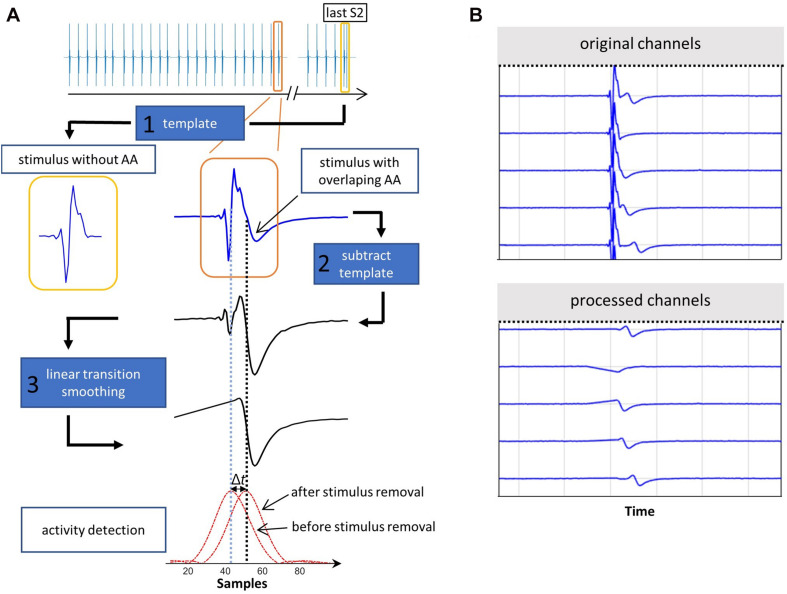
**(A)** Elimination of stimulation artifact. Exemplary stimulation signal covering an atrial activity from an S1S2 pacing protocol (orange). Template (1, yellow) chosen from the last part of the S1S2 protocol. After template subtraction (2) and linear signal smoothing (3), the maximum of the NLEO activity detection signal (red), shifts by Δ*t* from the stimulation (vertical blue dotted line) to the now uncovered atrial activity (black dotted line). **(B)** A synthetic signal segment containing stimulations followed by atrial activities. In some cases, the atrial activity is covered by the stimulation (original channels). After the matched filtering method is applied, all atrial activities become visible (processed channels).

After all stimulus time segments were assigned either an S1 or S2 tag in the previous step, the atrial activities have to be segmented. Two main problems arise: (i) the stimulation peak is more prominent in amplitude and slope than the atrial activity; (ii) for some combinations of CV and electrode distance, the atrial signal overlaps with the stimulation peak, thereby making it indiscernible for most activity detection algorithms. Thus, a matched filtering method was implemented designed to find specific signal morphology embedded in noise. This methodology maximizes the signal-to-noise ratio (SNR) using a template of a known signal, which is correlated with the measured noisy signal. Short signal segments and morphological impulses can lead to filter artifacts and oscillations, motivating an adapted approach. We detail the key aspect of template choice and our 3-step matched-filter implementation sub pipeline (Figure 3) in the following.

To decide on a stimulation signal template, all detected stimulation segments are checked for an overlap with the previously detected QRS complexes to exclude those with ventricular far field overlap from step 2. In the second step, a stimulation signal template is chosen for each channel as the first available S2 stimulation signal starting from (and including) the shortest S2 coupling interval. In most cases, the S1S2 protocol continues until the ERP is reached. Thus, the time interval after the S2 stimulus with the shortest coupling interval either contains no atrial answer signal or, if stopped prematurely, the atrial answer has the highest time interval to the S2 stimulation artifact making it the best candidate for a “clean” stimulation template. In the third step, the amplitude of the signal template is scaled to individually match each stimulus of the channel signal. The template signal segment is cross-correlated with the channel signal and subsequently subtracted from the channel signal at the time point of highest correlation value. Finally, the time between start and stimulation peak in the initially detected stimulation time window (subsection 2.2.1) is linearly interpolated to avoid large derivatives between samples, which would distort the following atrial activity detection. The NLEO method (Schilling et al., 2009),

(1) $$E_{n}=x_{n}^{2}-x_{n+1}+x_{n-1}$$

is used to detect the atrial activity. The subscript *n* denotes the current sample of an input signal *x* and *E_n* is the non-linear energy operator output signal. Applying the previously defined threshold *k* to these signals yields time points of start, peak, and end of the atrial activity time window. The time segments in which the NLEO signal of the atrial activity does not exceed the threshold are defined as having no detected activity. This can be the case for measurements with loss of capture during the measurement.

#### ERP Estimation

The last segmentation step is the detection of the ERP. After ventricular far field exclusion, the ERP is determined by checking when the atrial activity to an S2 stimulus dropped below an empirically determined threshold of 20% of the mean amplitude of all atrial activities to S1 stimuli in the same pacing train. The mean of the S1 stimuli inside a pacing train is considered since, in clinical practice, slight catheter shifts due to respiration and muscle contraction can lead to amplitude changes in the signal. The ERP is detected for the stimulating as well as the non-stimulating catheter.

#### Processing of Location Data

The location of each electrode per time sample is extracted from the clinical EP system. Since the pipeline uses bipolar signals, the mean location of the two electrodes is taken as a surrogate for bipole location. Inter-bipole distances between catheters are calculated using the geodesic distance by searching the shortest path on the mesh and subsequent Bézier spline interpolation (Naber et al., 2020). For the electrode distances within a single catheter, the Euclidean distance is chosen, since often the surface mesh is too coarse to compute mesh-based distances, and additionally, the projection onto the mesh results in non-negligible errors.

#### Amplitude and CV

Amplitudes are determined within the previously detected active segments and are calculated by subtracting the minimum from the maximum amplitude of the signal (peak-to-peak voltage).

The LATs of the atrial activities are known, and the distance between stimulation (*r_s*) and measuring electrode (*r_m*) is known. If an atrial geometry with sufficient LATs is given CV, Anisotropy and fiber direction can be estimated (Roney et al., 2019). To calculate CV from an electrode, existing method such as the ellipse fit (Blauer et al., 2014), the cosinus fit (Weber et al., 2011) or the CV fit for multiploar catheters (Roney et al., 2014) can be applied. These methods create a single restitution curve for the substrate at the specific measurement location by combining all LATs with the catheter geometry. However, most approaches are dependent on certain measurement setups or specific catheter orientation to the wavefront, which might not always be given for all S1S2 protocols. Thus, we use the general velocity equation from classical mechanics to showcase the CVAR-pipeline:

(2) $$p\,\left(t\right)=\frac{∥\vec{r_{s}}-\vec{r_{m}}∥}{L\,A\,T}$$

This is not the true CV of the depolarization wave but rather the scalar projection of the CV vector onto the path between stimulation and measuring electrode. We refer to this as propagation speed (p). Since the pipeline provides all spatial and temporal values, it is well suited to provide input to any other CV estimation method as well.

### Pipeline Performance Testing

To evaluate the performance of the pipeline with respect to noisy measurements, synthetic S1S2 protocols were built comprising stimulus peaks, atrial activities with rate dependent changes, baseline wander, and noise existing in clinical data.

#### Synthetic S1S2 Protocol Setup

**FIGURE 4:**
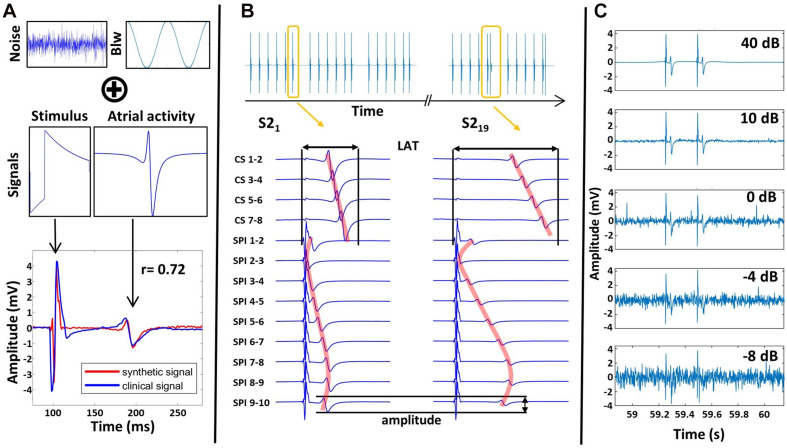
**(A)** Clinical noise and baseline wander (Blw) are combined with synthetic stimulus and atrial activity signals to create a synthetic signal close to clinical signals with the Pearson correlation coefficient *r* of 0.72. **(B)** Complete synthetic S1S2 protocol setup with multiple pacing trains (yellow), catheters, and catheter channels, anisotropic wave propagation (red) as well as amplitude & LAT changes due to restitution. **(C)** Effect of noise scaling on the signals.

Stimulus signals were modeled as exponentially decaying biphasic pulses (Figure 4A) following the formulation of Vigmond et al. (2003). The amplitude of the stimulation artifact was set to 4.7 mV as observed in our patient measurement. The total stimulation signal length was set to 8 ms, with the first part of the biphasic signal being 2 ms in accordance with the clinical stimulation setup. Atrial activities were modeled following Corino et al. (2013) based on a moving dipole in an infinite homogeneous conductor. The atrial activity synthetic signal morphology was scaled to have an amplitude of 2 mV and duration of 35 ms (Figure 4A).

Using these activity templates, a complete S1S2 protocol was created with an S1 BCL of 600 ms and an Δ*t*_*S*2*S*1_of 1.2 s. The S2 coupling interval was decreased from 500 ms to 350 ms in steps of 50 ms and from 340 ms to 180 ms in steps of 10 ms, yielding 21 pacing trains. The ERP was assumed to be 200 ms. Therefore, the last two pacing trains generated no atrial activity.

The S1S2 protocol was set up to mimic a clinical dataset for a circular catheter with a radius of 10 mm and 10 equidistant electrodes on a plane creating 9 neighboring bipolar signal channels. 4 bipolar coronary sinus (CS) catheter channels were set up with 35, 40, 45, and 50 mm distance to the stimulating channel. The stimulation was set up at channel 1 corresponding to the bipolar electrode pair 1–2. The LATs of the atrial activities in the channels were assigned, assuming elliptic excitation conduction with an anisotropy factor of 1.5. To mimic restitution behavior, an exponential decay of amplitude and CV was defined for the atrial activities of S2 stimuli (Supplementary Material section “Atrial Activity Segmentation”), starting from 2 mV for amplitude and 650 mm/s for CV (Figure 4B red line).

Different LATs across channels resulted in different levels of overlap with the stimulation signal. The overlap was determined by subtracting the LAT of the atrial activity template from the temporal end of the stimulation template in the global signal protocol. Channel 2 had an overlap of 51%, channel 1 46%, channel 3 48%, and channel 4 10% between atrial activity and stimulation. All other channels had no overlap (Figure 4B).

#### Synthetic Noise Generation

To represent noise, segments containing no atrial activity from clinical recordings were extracted. From these, a signal with equivalent frequency components was reconstructed using the discrete inverse Fourier transfer function. Additional baseline wander was implemented following Lenis et al. (2017), by adding frequency components *f_n* ranging from 0.001 to 5 Hz with uniform random distributed phases and amplitude of twice the magnitude of the atrial activity (Figure 4A). To test the limits of the pipeline, noise was added successively to the synthetic signal. This was implemented by amplification of the established noise time domain signal with a noise level factor *n*. This factor scaled the signal to the desired SNR power ratio according to

(3) $$n=10\,\left(\frac{P_{s\,i\,g\,n\,a\,l}}{P_{n\,o\,i\,s\,e}}-S\,N\,R\right)\,\frac{1}{20}$$

*P* is the power of the signal for the signal and the noise, respectively (Figure 4C).

### Evaluation

A single stimulation protocol with 9 channels combined with 19 S2 stimuli with decreasing coupling interval (the ERP is reached for lower S2 coupling intervals) led to 171 signals. Exclusion of the stimulation channel yields 152 signals and thus the same number of detectable LAT values. The measure of error was the deviation of the respective pipeline results (number and value of LAT, amplitude) from the ground truth.

To evaluate the performance of the pipeline under noise-free conditions, the restitution curves of amplitude and propagation speed were compared against the ground truth used to create the synthetic signals. We report conduction speed since we computed the scalar projection of the CV vector onto the path between stimulation and measuring electrode.

To evaluate the overall change of accuracy for decreasing SNR levels, the median of all 152 values was used. Additionally, the 25th (Q1) and 75th (Q3) quantiles of deviation from the ground truth were computed across all channels and all S2 stimuli. We plot the interquartile range here since no outliers exist for the clinical SNR range after exclusion of channel 2 which was used to test the absolute limits of the pipeline in regards to overlap between stimulation artifact and atrial activity (Chapter 2.3.1). The boxplot version of the figure can be found in “LAT Error on Synthetic Data” of the Supplementary Material. Since the median does not account for missed LAT detections, the number of atrial activities which were not detected but were present in the synthetic signal was evaluated as well.

### Clinical Data

The proposed pipeline was additionally tested on one clinical dataset recorded in the EP department of Städtisches Klinikum Karlsruhe. The patient (59 years) presented with paroxysmal AF. The S1S2 stimulation was carried out with a 20-pole circular catheter on the posterior atrial wall. The signals were stored in the Bard recording system (Boston Scientific, Marlborough, MA, United States). The atrial geometry was recorded with the CARTO EP mapping system (Biosense Webster, Irvine, CA, United States). Data were anonymized and exported for further investigation. The study was part of a standard ablation procedure and approved by the ethics committee. The patient gave informed written consent.

LATs from the clinical signals acquired from the S1S2 protocol were manually annotated by two independent electrophysiology experts for evaluation of the tool. Furthermore, morphological, and physiological qualitative accordance with literature data was assessed for both amplitude and propagation speed restitution curves in lack of ground truth data. For the interested reader LAT restitution was calculated as well and is available in “LAT Restitution” in the Supplementary Material.

## Results

### Synthetic Signals Faithfully Reproduce Clinical Electrogram

To test the pipeline with a known ground truth, synthetic signals resembling clinical recordings were created as described above. Similarity of clinical and synthetic signal was assessed by evaluating the Pearson correlation on a signal excerpt of one channel for each noise realization and was found to have a maximum at 40 dB (*r* = 0.72). Decreasing SNR (Figure 4C) below 40 dB decreases similarity with the clinical signals down to 0.17 for −10 dB. At 0 dB, mean noise amplitude reaches a third of the atrial activity amplitude, which is seldomly seen in clinical bipolar measurements. For even lower SNR values, noise peak amplitude is equal to or surpasses atrial activity amplitude.

### Valid Reproduction of True Propagation Speed and Amplitude From Synthetic S1S2 Protocol

The temporal histogram approach consistently estimated the S1 BCL of the synthetic signals with an error below a single sample duration (1 ms). The same holds true for the statistical k-means clustering method used to detect the number of pacing trains and pacing stimuli contained in the single pacing trains. These two segmentation steps build the foundation for all future segmentation steps and proved to be robust for all noise conditions.

**FIGURE 5:**
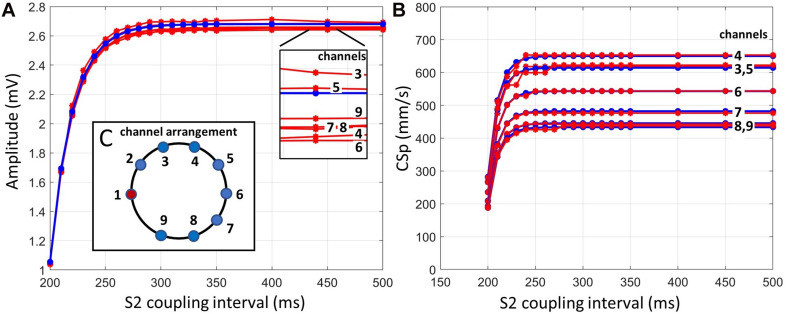
**(A)** Ground truth amplitude of atrial activity for all channels (blue) and pipeline outcome amplitudes (red). **(B)** Ground truth propagation speed (blue) and pipeline outcome propagation speed (red). Both panels represent an almost noise-free case (SNR 40 dB). **(C)** Schematic sketch of bipolar channel arrangement mimicking a clinical case resulting from 10 electrodes.

The ground truth atrial amplitudes were compared to the amplitude curves reconstructed by the pipeline and showed a maximum deviation of 0.05 mV (Figure 5A).

The estimated propagation speed values (Figure 5B) resulting from the pipeline revealed channel 5 to have the largest error with a mean deviation of 7.81 mm/s (1.3%), originating from an LAT error of approximately 0.34 ms across all data points. Channel 3 shows a single deviation of 35.2 mm/s (5.7%) at a coupling interval of 230 ms, resulting from an LAT error of 0.7 ms.

The LATs of the channels were set up to create different levels of overlap of the atrial signal and the stimulation peak (Figure 4B). The pipeline was unable to detect the atrial signals where the atrial activity overlapped more than 50% with the stimulation artifact, which was the case for channel 2. All other atrial answers were detected. However, channel 1 was excluded since it was defined as the stimulating channel. Channels 3 and 5 had the same propagation speed restitution curve due to the circular geometry and the LAT model and only differed in noise to test the consistency of the algorithm in later noise evaluations.

### Valid Reproduction of Propagation Speed and Amplitude Beyond Clinical Noise Levels

**FIGURE 6:**
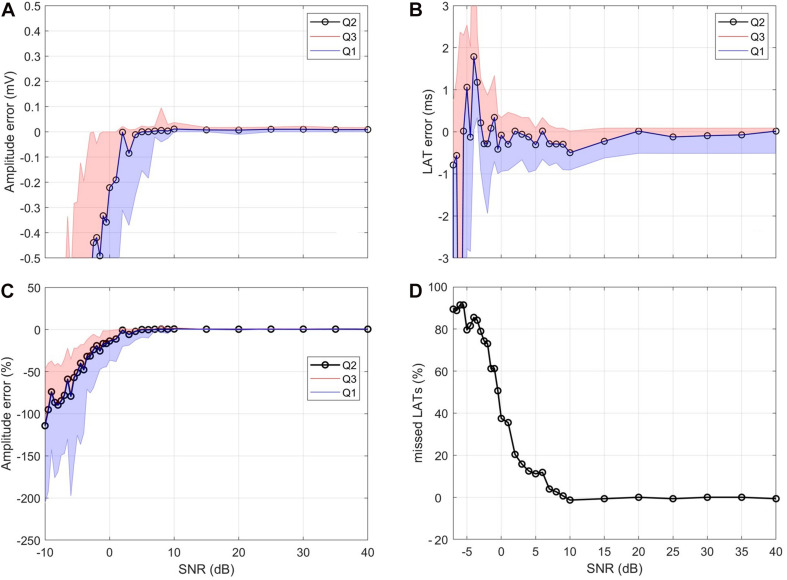
Influence of noise. **(A)** Median (Q2) amplitude error and interquartile distance (Q1, Q3). **(B)** Median LAT error and interquartile distance. **(C)** Relative amplitude error with respect to true amplitude. **(D)** Percentage of missed LAT detections. For **(A,B)** the *y*-axis was capped for visualization purposes.

The reconstructed amplitude for synthetic signals with decreasing SNR (Figure 6A) showed a stable mean amplitude error of 0.06 mV down to 10 dB. Below 10 dB, there was a marked increase in the interquartile range, and the median value dropped. At 0 dB, the median error exceeded 0.2 mV. The amplitude error relative to the true value exceeds 10% at 1 dB and 20% at −1.5 dB (Figure 6C).

The median LAT was stable with errors below a single sample down to −1 dB (Figure 6B). The median value began to oscillate within the 1 sample error margin for values below −1 dB. For values lower than −3 dB, interquartile distance drastically increases, and the median oscillates by values larger than one sample.

Figure 6D shows the number of atrial activities in the synthetic signal which were not detected. Above 9 dB, the number of failed detections was below three resulting in relative errors close to 0%. For lower SNR levels, the number of failed detections increased up to a value of 142, resulting in relative errors close to 90%. These values exceed the range of clinically valid measurements and are used to test the limits of the pipeline.

### Plausible Restitution Curves for Clinical Signals

**FIGURE 7:**
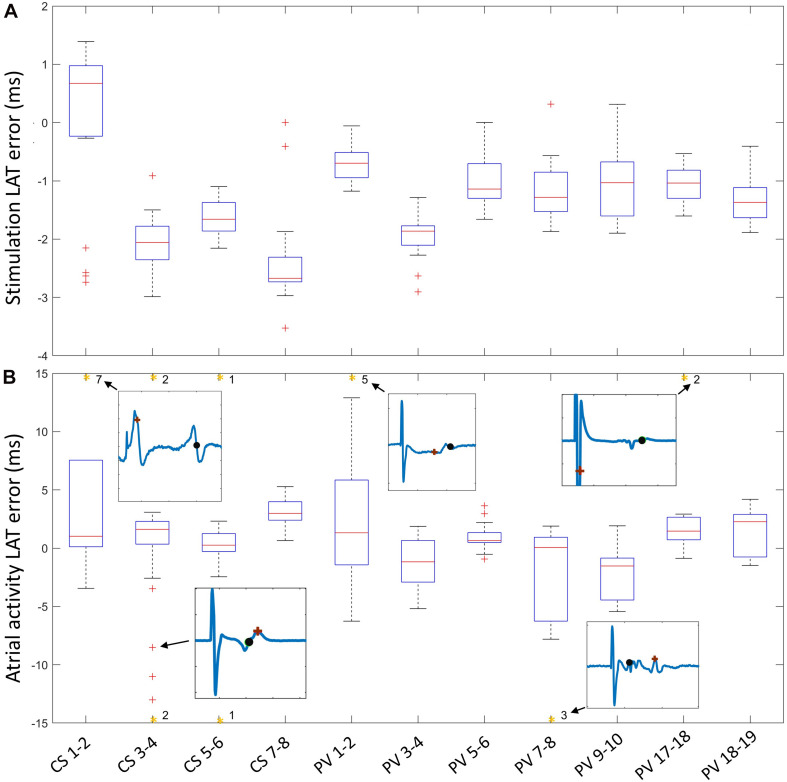
LAT error of stimulation signal **(A)** and atrial activity **(B)** calculated by subtraction of pipeline LAT from expert annotation. The number of outliers beyond the *y*-axis limit (orange asterisk) is shown, along with an exemplary signal showing the differences between the expert annotation (black) and the pipeline annotation (brown).

The LAT error was created by subtracting LATs resulting from the pipeline from the manual expert annotations. LAT errors of the stimulation signal remained in the range of −4 ms to 1.5 ms (Figure7A). CS LAT error had a larger variance in median value between channels while the median LAT error in the circular catheter remained approximately −1 ms for all channels except PV3-4 (1.9 ms). Atrial activity error (Figure 7B) median values remained in the range between −1.5 ms and 3 ms for all channels (Figure 7B). Interquartile ranges were below 3.5 ms for all channels except CS1-2, PV1-2, and PV7-8, in which the interquartile range was approximately 7.5 ms. Asterisks at the upper boundary of Figure 7B denote the number of extreme outliers beyond the *y*-axis limits for each channel. In total, 23 (7%) out of 321 LAT errors were considered extreme outliers. Excerpt signals of the extreme deviations show the expert annotation (black) and pipeline results (brown).

**FIGURE 8:**
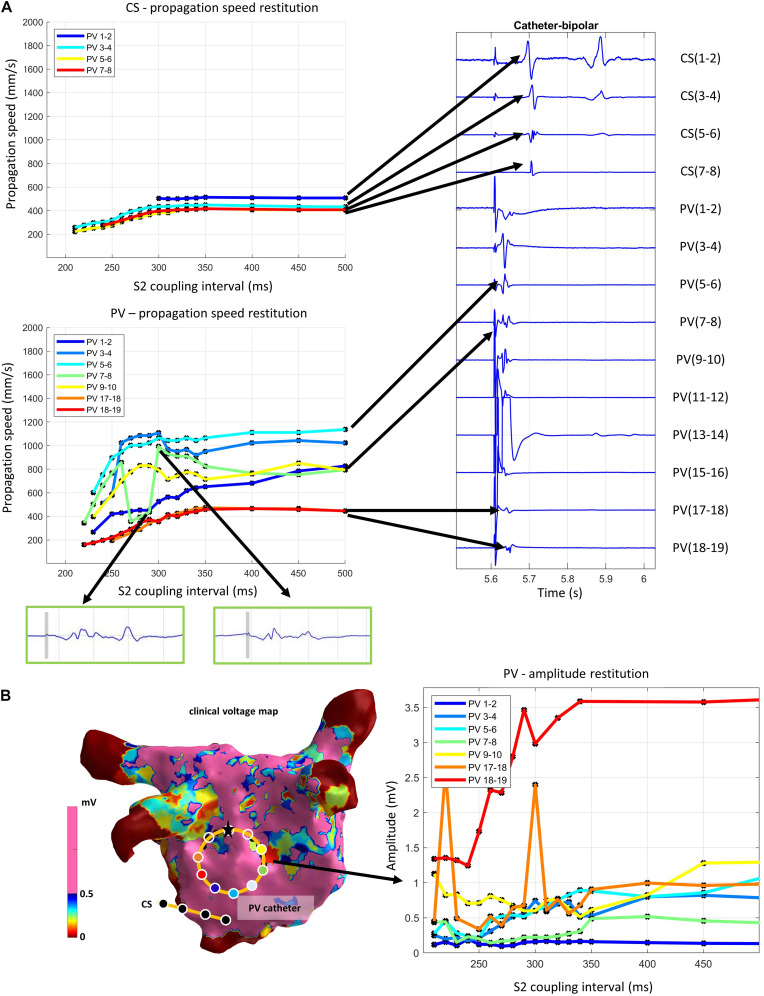
**(A)** Exemplary output of the pipeline applied on a clinical measurement with stimulation from the circular catheter electrodes 13–14. Propagation speed restitution curves of each channel of the CS and the circular (PV) catheter (left) and the first corresponding S2 signal segment (right). Green boxes show excerpts of atrial activity signal after removal of the stimulation artifact (time segment marked in gray). **(B)** Voltage map (left) with circular catheter (PV) with white circles showing bipolar channel positions. Reconstructed amplitudes of circular catheter (stimulation from electrodes 13–14) with black dots representing data points (right). Lines are linearly interpolated in between. Line colors correspond to the color of the dot of the channel position on the voltage map.

Figure 8A shows an exemplary output of the pipeline when applied to a clinical measurement with stimulation from the circular catheter (PV) electrodes 13–14. The reconstructed propagation speed restitution curves in the spatially distant CS catheter (Figure 8A top) showed a smooth morphology. The propagation speed asymptote ranged from 407 to 510 mm/s. In the circular catheter (Figure 8A bottom), the channels neighboring the stimulation channel (11–12, 15–16) are removed by default by the pipeline. For all other channels, the stimulus directly transitioned into the atrial activity (Figure 8A right). The atrial activity, however, remained distinguishable from the stimulation. The automated removal of the stimulation signal delivered plausible results for clinical measurements inside the stimulating catheter, as shown by the morphology of the restitution curves. A jump can be seen in PV 7-9 (green curve) at 300 ms. This is due to prolonged atrial activity (green box), where at one instance, the peak nearer the removed stimulation (gray bar) is detected at the next point the further peak is detected, creating a jump in propagation speed.

The clinical voltage map (Figure 8B left) with the highlighted catheter position indicated that the measurement took place in a region of normal voltage. Most amplitude restitution curves have an asymptote of approximately 1 mV, thus above the prevalent clinical threshold for low voltage of 0.5 mV. The curve PV 7-8 has an asymptote around 0.5 mV. PV 1-2 is the only curve seemingly without restitution morphology. Since the absolute amplitude of the whole channel measurement was consistently lower than all other channels, the morphology is most likely a result from insufficient atrial wall contact due to rigid catheter design. The amplitude measured by the circular catheter follows a restitution pattern with the largest decay between 350 and 330 ms S2 coupling interval (Figure 8B right). This is in accordance with the drop of propagation speed at 350 ms S2 (Figure 8A). The amplitudes for shorter S2 intervals varied strongly for 2 of the 6 channels.

## Discussion

This study presents and evaluates a fully automated signal segmentation pipeline for the S1S2 protocol. The pipeline has 3 guiding requirements: (i) No a-priori knowledge of the measurement should be used, and all evaluations should be derived purely from the input. (ii) All steps should be fully automated and yield precise results while remaining robust under noise conditions. (iii) It should handle the evaluation of the atrial activity in proximity to the stimulation artifact. The main result of this work is the openly available CVAR-Seg pipeline, which can accurately segment an S1S2 protocol and create amplitude and propagation speed curves under a wide range of noise conditions. The pipeline fills the gap of automatically evaluating restitution protocol information independently from mapping system, stimulus generator, catheters, and stimulation parameter setups. To the best of our knowledge, there is no other complete pipeline for evaluation of the S1S2 protocol or closely related stimulation protocols.

An LAT detection pipeline was proposed by El Haddad et al. (2013) where a clean reference channel is used for rough pre segmentation of activity, then ventricular far field is blanked, the signal is rectified, and the center of mass is calculated. We use the same fundamental idea by first identifying the stimulation peaks and using them as reference points to create time windows in which to assess the atrial activities. For our pipeline, regular far field blanking is not possible. Due to the changed pacing intervals, atrial activities can overlap partially with the ventricular far field segments and excessive blanking might erase large parts of the atrial activity in some pacing trains. The approach proposed by El Haddad et al. (2013) focuses solely on signal traces without stimulation artifacts and, in part, relies on unipolar signals which are not always available. Therefore, alternative methods were sought.

Cantwell et al. (2015) reviewed the most common methods for automated LAT annotation: morphological approaches, NLEO, time-delay cross-correlation, wavelet decomposition, deconvolution, template methods, and gradient methods. We used the two best performing signal segmentation algorithms, namely the wavelet and the NLEO method, as proposed by Lenis et al. (2016). The wavelet method relies on a mother wavelet which in turn necessitates some estimation of the signal. This can only be done for the stimulation signals. Using the bior1.5 as proposed by Lenis et al. (2016), we only assume a steep flank in the stimulation signal, which is always present in any stimulation and therefore does not violate our requirement of not using a-priori information. For atrial activities, no assumptions were made. Therefore, we used the wavelet to detect the stimulations and the NLEO method for the atrial activity. To determine non-detected activities, we used a NLEO threshold proposed by Lenis et al. (2016). The choice of threshold is a tradeoff between detecting more peaks with potentially increasing errors versus detecting less but more trustworthy peaks. We see a potential for improvement here by using an adaptive NLEO threshold based on estimation of the SNR of the signal as proposed by El Haddad et al. (2013).

Kremen and Lhotska (2007) proposed to detect signal components in different complex fractionated atrial electrograms (CFAE) classes based on the wavelet transform. This could be used to extend this pipeline to incorporate an evaluation of fibrillatory events and annotate atrial activities. An alternative approach could be to use the recently published openEP project (Marino et al., 2021), which enables EP data parsing and analysis.

Other works use manual (Corrado et al., 2017) or semi-automatic pipelines (Corrado et al., 2018; Abdi et al., 2020) to evaluate restitution curve morphology. The latter might be more proficient for an evaluation on the atrial surface mesh. The CVAR-Seg pipeline rather focuses on evaluating singular electrodes, and the evaluation would have to be projected on the 3D atria in a subsequent step.

CVAR-Seg works in a fully automated manner, can be used on large patient cohorts, and demonstrated applicability on clinical recordings. This was shown by evaluating 37 different SNR noise scenarios (55,278 synthetic signals) and an exemplary clinical case without manual interaction. While SNR of clinical signals can, in general, be estimated by comparing filtered versions of the signal, reliable estimates are hampered by different filter settings, clinical setups, and measurement quality. Our arbitrarily picked clinical measurement corresponded to a synthetic case of 40 dB and is assumed to be a good representation of a mean clinical noise level. The range was expanded by looking for the noisiest possible signals in literature. Visual comparison with literature (Verma et al., 2018; Unger et al., 2019; Jadidi et al., 2020) and available clinical measurements suggest a clinical noise level between 40 and 10 dB in terms of our synthetic setup (Figure 4).

Extra beats where accounted for in the detection of the pacing trains and during the evaluation of the single stimulus and following atrial activity time segments. In most cases the stimulation will create the depolarization wave and the tissue will not be activated by extra beats due to the ERP. Should an extra beat occur in the time window of evaluation it cannot be morphologically distinguished from a stimulation induced depolarization wave and must therefore be excluded during postprocessing. We suggest excluding points above threefold standard deviation of the curve. The proposed pipeline gives accurate results for amplitude, LAT, and consequently propagation speed (Figure 5) down to SNR values of 0 dB. If some misdetection of atrial activities is acceptable, the signal quality can even deteriorate to -4 dB (Figure 6). LAT evaluation errors remain mostly below a single sample and amplitude errors in the range of 0.06 mV. Down to 1 dB, the relative amplitude error is below 10%, which is deemed acceptable since other processing steps influence the amplitude more profoundly, e.g., density of sampling points, voltage map interpolation, and method of amplitude calculation in each EP system.

For SNR beyond the clinical range of 10 dB, the errors in amplitude and LAT increase markedly while, at the same time, the number of non-detected LATs rises. The LAT errors translate to larger errors in propagation speed. However, the median LAT value remains nearly constant for all SNR. This means we still obtain mostly correct values and several extreme outliers stemming from large noise peaks in proximity to the atrial activity in some channels. Signal processing solutions would most likely also impact atrial activity morphology. Another way these outliers could be mitigated is by using one of the fitting approaches incorporating all measured values (Weber et al., 2011; Blauer et al., 2014). The general underestimation of amplitude for SNR below 10 dB is due to high noise amplitudes causing the pipeline to mistake noise peaks for atrial activity. As noise grows larger in amplitude, the error trends toward more negative values due to the algorithm increasingly overestimating the amplitude. The increase in spread is then due to different noise realizations in the different channels. For extreme SNR cases below −4 dB, we observe noise amplitudes of equal magnitude as the atrial activities with occasionally noise peaks higher than atrial activity amplitude. Evaluation of such measurements necessitating adjustments of the default filter settings of the pipeline.

### Outlook for Clinical Data

Comparison of expert annotation LATs against the pipeline produced LATs revealed good accordance for the stimulation signal. Most LATs from the circular catheter channel median values were merely deviating by −1 ms from the expert annotation. The larger shift in median (−2 ms) present in the CS catheter channels. The large variability can be explained by the fact that during stimulation in the spatially distant circular catheter the stimulation presents as far field in the CS catheter channels with minimal amplitude and slope due to the bipolar arrangement, and signal noise around the stimulation artifact incurred small deviances in the non-linear energy operator peak.

The median atrial activity error with an error of 0 ms is a promising result. Interquartile ranges of most channels resulted in minimal, but acceptable shifts in LAT since all LATs remained within the atrial activity. The different annotation styles (maximum and minimum peaks or maximum slope) between experts explained the larger interquartile ranges of CS1-2, PV1-2, and PV7-8. The extreme outliers reveal that deviations occurred due to several reasons: (i) different annotation between pipeline and manual evaluation; (ii) ventricular far field overlap with the stimulation in the CS catheter. For time windows that contain a stimulation, no far field removal was performed since, in many cases, the bipolar signal would reduce the ventricular far field enough for evaluation, and exclusion of all stimuli overlapping with ventricular far field could result in deletion of too many time segments. (iii) incomplete removal of stimulation artifact leading to premature LATs; (iv) extended atrial activities. These cases accounted for 7% of all annotations, leading to the conclusion that the pipeline yields adequate results. Outliers produced by extreme cases can be filtered out in a postprocessing step since they will be deviating from the restitution morphology of all other points.

The segmented restitution curves from the paroxysmal AF patient showed the following properties: All catheter channels show different asymptotes of propagation speed curves, which is expected due to fiber orientation-induced CV anisotropy. Using the minimum and the maximum propagation speed curves as a first estimate, the resulting anisotropy ratio of 3.4 is in accordance with experimental reports ranging up to anisotropy levels of 5 and above (Spach et al., 1988; Harrild and Henriquez, 2000; Roney et al., 2019). With a mean asymptote of 692 mm/s, our propagation speed lies within the reported ranges values for experimental data ranging from 100 mm/s (Spach et al., 1988) to 1800 mm/s (Roney et al., 2019) and is close to the value of 600 mm/s reported in Verma et al. (2018). Global CV for AF patients has been reported in a range of 511 ± 110 mm/s (Zheng et al., 2017) in line with our asymptote measurement of 439 mm/s between the circular catheter on the inferior posterior wall and the CS catheter. Recent works extend CV estimation by including LAT measurement uncertainty (Coveney et al., 2020) which could potentially be incorporated into CVAR-Seg in the future. Roney et al. (2021) extend the concept of scalar CV by estimating atrial fiber direction along the endocardial surface. Using CVAR-Seg, this method could be applied to restitution data to see if preferential conduction directions stay consistent when derived from CVs acquired at different pacing rates.

Local amplitude restitution ranges from approximately 1 mV to below the clinically used low-voltage threshold of 0.5 mV (Jadidi et al., 2016) for each spatially stable measurement. Thus, the amplitude decrease stems from restitution behavior of the tissue, not catheter roving. This also demonstrates that the single threshold used for voltage guided ablation might not be optimal for all patients since they present with different and highly variable heart rates due to AF (Nairn et al., 2020).

The clinical voltage map shows that our measurement was done in a region with normal voltage associated with healthy tissue. We observed a clear exponential morphology for both amplitude and propagation speed, which is in line with the exponential restitution curve morphology of healthy tissue reported in literature (Kim et al., 2002; Weber et al., 2011).

### Limitations

In the scope of this work, not all signal morphology varieties that can arise clinically due to different hardware (e.g., electrode dimensions, catheter spacing) and software (filtering values, filtering algorithms) setups could be covered *in silico*. Therefore, we considered the most important cases including restitution of amplitude and propagation speed, overlap of atrial activity with stimulation, and clinical noise variation. Since a guiding principle was to not use any a-priori information, the pipeline is not limited to the shown synthetic signals and does not exclude signals that deviate from our setup.

For pacing train detection, the k-means algorithm requires markedly longer intervals between pacing trains than the S1 cycle length. For values lower than 1.2 times the S1 cycle length the k-means clustering can yield unreliable results. A value of twice the S1 cycle length yielded correct clusters for the clean signal setup as well as a setup with noise and extra beats. For suppression of the stimulation signal, our matched filtering approach gives accurate results provided the depolarization slope of the atrial signal is not more than 50% covered by the stimulation. This creates a temporal lower boundary on detectable atrial activities, which is in large parts due to the linear smoothing of the first part of the stimulation time segment. This smoothing step is necessary to erase stimulation parts that cannot be erased by the subtraction of the template and becomes increasingly important when dealing with noisy signals and cannot be removed. In the future, other methods could improve detection of the atrial activities and dispense with the smoothing step, thereby lowering the temporal detection threshold further. One such approach could be the Changepoint method (Killick et al., 2012), which might achieve better detection results for fractionated signals. Additionally, other signal segmentation methods using statistical or machine learning approaches could prove beneficial for a better atrial activity segmentation and further improve robustness and lower the number of misdetected LATs for high noise cases.

### Conclusion

In this work, we provide the noise-robust signal segmentation pipeline CVAR-Seg for the widely used S1S2 stimulation protocol. It enables automatic computation of amplitude and propagation speed restitution curves from clinical data. The pipeline is built for S1S2 stimulation protocol measurements. However, the methodology could easily be transferred and adapted to any other stimulation protocols used in EP studies since the main problem of proximity between stimulus signal and atrial activity remains the same. At the same time the pipeline components are modular and can be easily replaced by alternative methods according to the user’s needs. This tool allows for a fast and precise evaluation of large datasets and eliminates the need to analyze each dataset manually. The proposed CVAR-Seg pipeline could serve as a basis for a standardized way of evaluation fostering reproducibility and comparability of future restitution studies.

## Data Availability Statement

The datasets presented in this study can be found in online repositories. The names of the repository can be found below: https://github.com/KIT-IBT/CVAR-Seg.

## Ethics Statement

The studies involving human participants were reviewed and approved by Institutional Review Board of Freiburg University, Freiburg, Germany. The patients provided their written informed consent to participate in this study.

## Author Contributions

AxL, GS, OD, CS, LU, EW, and MN conceived and designed the study. MN implemented and tested the pipeline. ArL and AJ acquired patient data. JS and MN implemented and tested the synthetic noise model. MN was responsible for creating the manuscript. All authors critically revised the manuscript.

## Conflict of Interest

The authors declare that the research was conducted in the absence of any commercial or financial relationships that could be construed as a potential conflict of interest.
