# Supplementary material for: CVAR-Seg: An Automated Signal Segmentation Pipeline for Conduction Velocity and Amplitude Restitution
Source: Front Physiol. 2021 May 24;12:673047. doi: 10.3389/fphys.2021.673047 (PMC8181407; doi:10.3389/fphys.2021.673047)
Supplement: Supplementary file 1 [file Data_Sheet_1.PDF]

Supplementary Material

1 Supplementary Data

1.1 Supplementary Figures

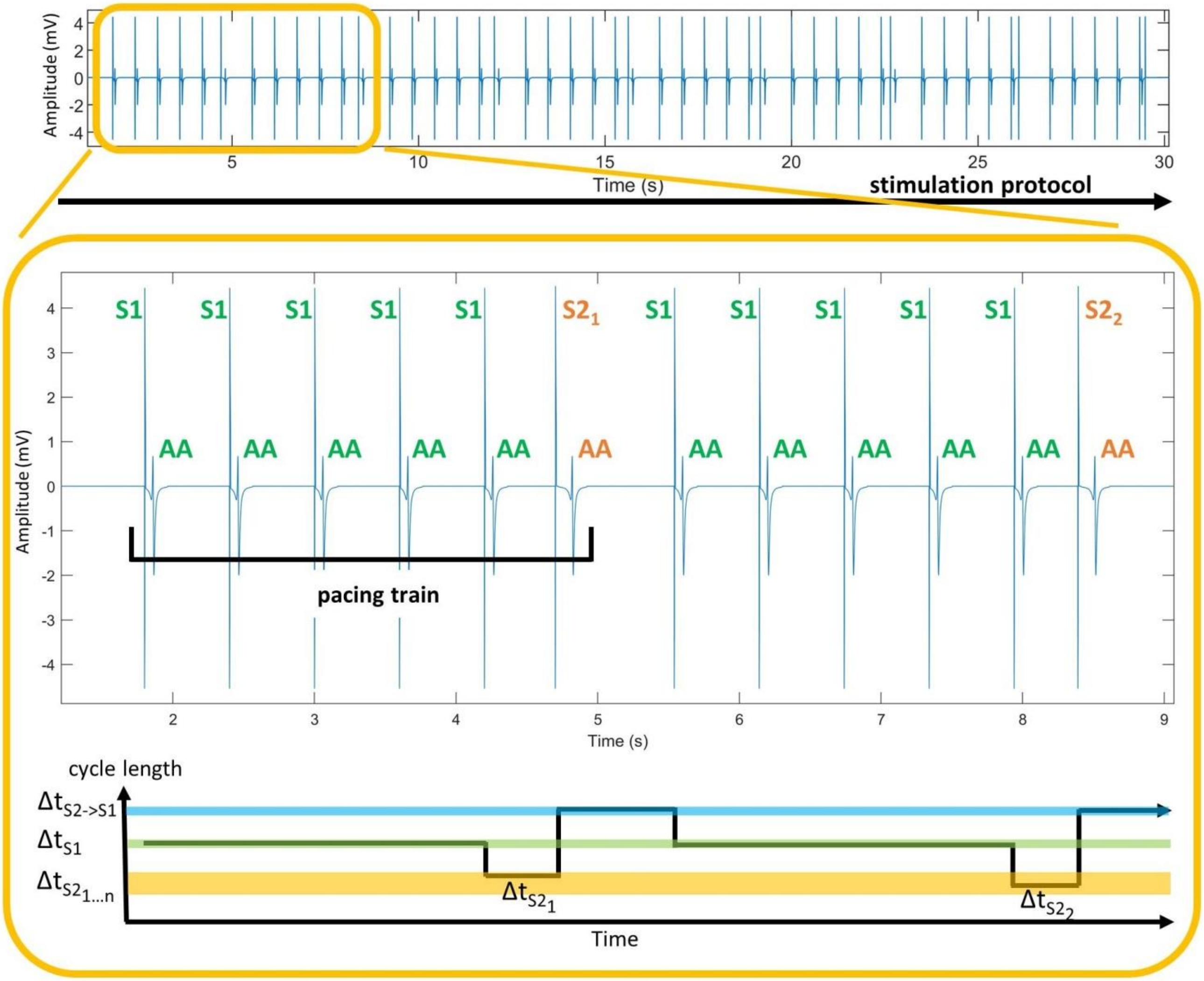

Figure 1**:** *An exemplary S1S2 protocol electrogram consisting of 5 equidistantly spaced S1 stimuli (green) followed by premature S2 stimuli (orange) with a smaller time interval Δt_{S2i}. Each successful stimulus is followed by an atrial answer (AA). A sequence of multiple S1 (5) and a single S2 stimulus is referred to as a 'pacing train'. The whole stimulation protocol consists of multiple repetitions of pacing trains with iteratively decreasing S2 coupling intervals (Δt_{S2i}). The black curve at the bottom depicts the three distinct time intervals in each train of a standard S1S2 protocol.*

Each pacing train contains several stimuli with a BCL, called the S1 stimuli, followed by a single stimulus which is administered after a reduced coupling interval, called the S2 stimulus. This pacing train is repeated, with the S1 stimuli retaining their BCL and an incrementally reduced coupling interval between the last S1 stimulus and the S2 stimulus. The pacing protocol ends as soon as the atrial tissue does not capture the stimulus anymore at a certain S2 coupling interval because it falls below the effective refractory period (ERP). In clinical practice, stimulations are applied by a stimulus generator connected to the electrophysiological recording system. The system directs the stimulus to the desired catheter electrode pair, which has been inserted into the atria of the patient.

1.1.1 Number of pacing blocks and stimuli estimation

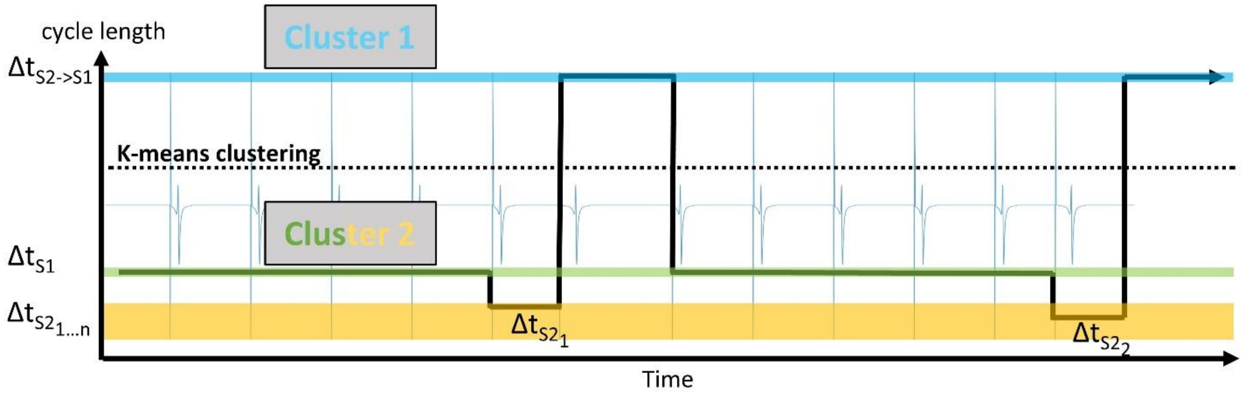

Figure 2: *Estimating number of pacing trains. Applying k-means clustering with two clusters on three main time intervals between stimulations yields pacing trains.*

1.2 Detection of stimulus time segments

For optimal stimulus time segment detection multiple factors had to be considered and weighted appropriately. In the following we will give the conditions posed on each detected stimulus time segment and the weights resulting from passing that condition. The weight of a single stimulation time segment W_{ts} was calculated using:

$$\boldsymbol{W_{ts} = n_{sc}(\alpha_s + \beta_s) + n_{nsc}(\alpha_{ns} + \beta_{ns}) + \gamma + \delta + \epsilon\,.} \quad \textbf{(1)}$$

n_{sc} is the number of bipolar stimulation channels which is 1 and n_{nsc} the number of non stimulating channels which in our case is 8. The first condition posed on stimulation time segments is that the activations are detected in multiple channels of the catheter at the same time. A detection in the stimulation channel gives a weight α_s of 2 and in the non-stimulating channel a weight α_{ns} of 1. The second condition was that the peaks detected with the high-pass filtering overlapped with the

detected stimulation time segments. The weighting factors β_s and β_{ns}were 20 and 10 respectively. The third condition was that the when creating the first principal component (PCA) of all channels, peaks are detected in the same time segments and surpass an amplitude threshold defined as 90 % of the maximum first PCA amplitude. The weighting γ added for passing this test was 100. The next condition was the time interval between neighboring stimuli. If they did not deviate from the S1 cycle-length by more than 10 samples the weight δ of 100 was added. The final condition is that after applying a PCA of all derivatives of all channel signals the first PCA component during the stimulation segments had to surpass 90 % of the first PCA signal amplitude which yields a weight of 100 for ϵ. The resulting sum of weights for each detected time segment can be seen in Figure 5.

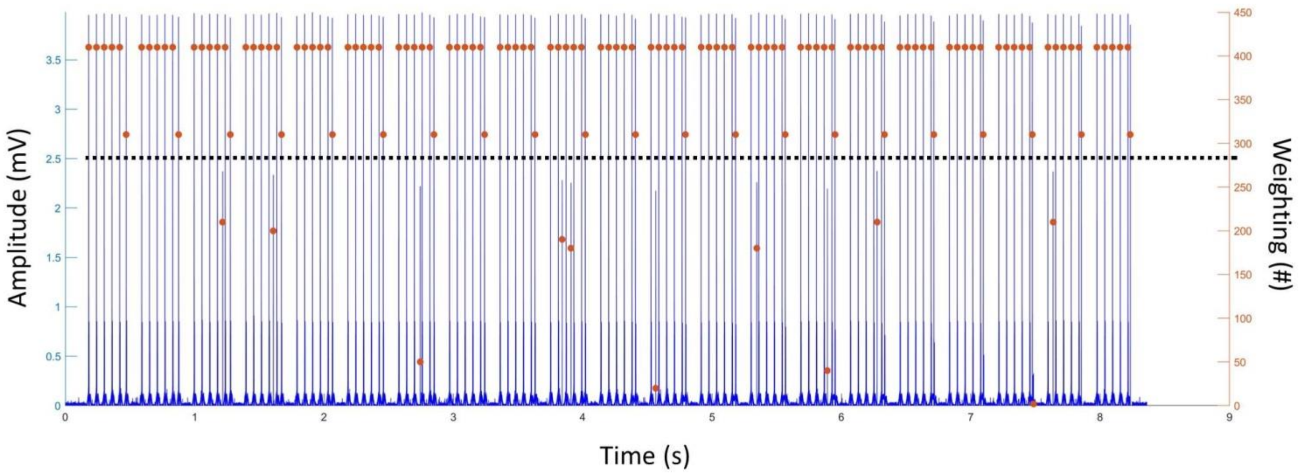

Figure 3: *Each peak of an S1S2 protocol (blue) acquires a weighting (orange). Peaks with high weight are stimulation peaks (here: weight>300). Detected peaks with lower weight correspond to extra beats. Threshold (black dotted line) for peak acceptance was set at 277.*

The weights are used to deduce trustworthy stimulations from extra beats and noise. In a final step, a threshold of the previous weighting is performed. The threshold was set so that a stimulation had to be detected in at least half of the 9 channels, had to pass the amplitude and derivative test leading to a threshold of 277 (5•1+1•2+5•10+1•20+100+100). Applying the threshold divids the peaks into trusted stimulation time segments and ignored stimulus time segments. The remaining time segments need not all have the same weighting for all peaks. Therefore, four possible weight distribution outcomes of the remaining trusted peaks were implemented:

i) In the ideal case, there are exactly 2 weights left. All S1 stimuli have the highest weighting and all S2 peaks with a lower (-100) weighting, since they fail to pass the S1 cycle-length interval test. In this case S1 and S2 stimuli can be distinguished from each other and segmented.
ii) Most stimulus time segments have the highest weighting and some stimuli have a lower weighting and vary in weight. If the number of time segments with the highest weight fits the expected number of S1, which was calculated using the information of S1 time and number of pacing trains earlier, S1 and S2 time segments can be segmented by assigning the highest weight as S1 and all other weights as S2 time segments.
iii) Most stimuli have a high weight and vary in weight but the number of time segments with the minimum weight corresponds to the number of expected S2 time segments, calculated

earlier. In this case S1 and S2 time segments can be segmented by assigning the lowest weight as S2 and all other weights as S1 time segments.

iv) The remaining stimulation time segments have multiple weights and neither maximum weight correspond to the expected number of S1 nor does the minimum weight correspond the expected number of S2, making a distinction by weight alone impossible. If the combined number of remaining stimulus time segments is correct but the weights do not allow for segmentation into S1 and S2 stimulus by means of i), ii) or iii) due to electrodes having no signals or other measurement related deviations (morphological or amplitude wise), then a counter is used to assign the S1 and S2 tag to the stimulus time segments.

If none of the two cases are met, a reconstruction algorithm is used based on a mean pattern recognition theme. This is done by constructing a mean template pattern of a single stimulation train consisting of the expected number of S1 stimuli, determined in a previous step, followed by a single S2 stimulus. Stimuli were modeled as Gaussian bells with a width of the mean stimulus time segments length and amplitude of 1. The whole template is cross correlated with a step function of the signal where the stimulus time segments where 1 and the rest of the signal 0. The maximum of the cross correlation yields the best fit of all stimuli in the pacing train with the signal and all stimulus segments can be assigned S1 and S2 tags. This concludes assignment of S1 and S2 to stimulation time segments.

1.2.1 Atrial activity segmentation

Additionally, an exponential decay of amplitude and of LATs for a circular catheter was defined:

$$\boldsymbol{y_{restitute} = -a \cdot e^{-b \cdot (t_{s2} - t_{ERP})} + c} \ . \qquad \textbf{(2)}$$

where a was 2 mV for amplitude and 1000 mm/s for CV, b was 0.05 mV for amplitude and 0.1 1/ms for CV, t_{s2} were the S2 intervals, t_{ERP} was the S2 coupling interval where the ERP was reached and c was 2 mV for amplitude and 650 mm/s for CV.

1.3 LAT restitution

Figure 4 shows the LAT restitution for different catheter channels.

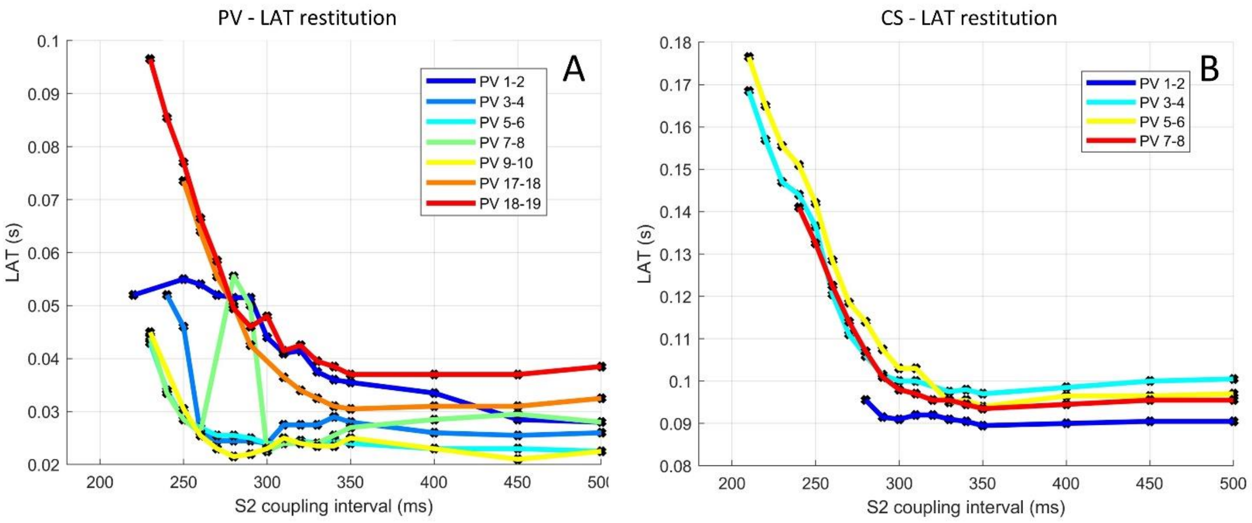

Figure 4: *Exemplary output of the pipeline applied on a clinical measurement with stimulation from the circular catheter electrodes 13-14. LAT restitution curves of each channel of the circular (PV) catheter (A) and the CS catheter (B).*

1.4 LAT error on synthetic data

LAT errors larger than q3 (Figure 5A) occur and the LAT error table (Figure 5B) shows they originate from channel 2. This channel was used to test the limits of overlap between stimulation and atrial answers that can be reconstructed. The pipeline was set up with different overlaps and it was unable to detect the atrial signals where the atrial activity overlapped more than 50 % with the stimulation artifact, which was the case for channel 2.

All other relevant results reconstructed LATs lie within q1 and q3 down to -5dB. For lower SNR (<-5dB) levels outliers originate from other channels as well. These values are extreme cases and would seldomly occur in clinical measurements or be used as study data. For high noise (SNR<-5dB) it is impossible to reconstruct the LATs and the authors recommend using this tool with signals down to 0dB.

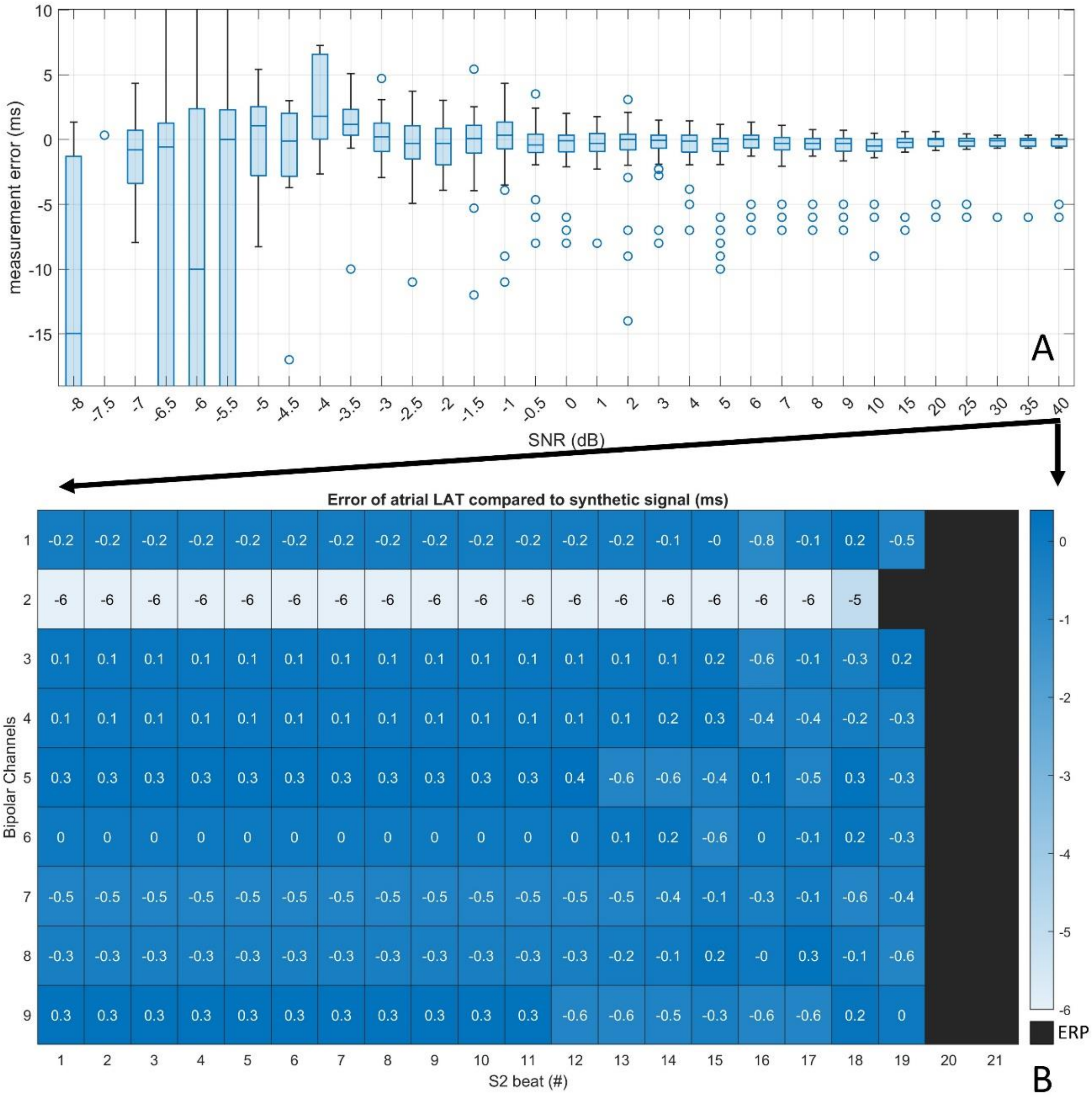

Figure 5: *Influence of noise. Amplitude error of LATs (A) and a table showing the LAT error of all channels and all S2 pacing trains of the 40 dB case (B). Numbers refer to the LAT error value and black boxes depict no atrial activity detection. Outliers (circles) in A originate from channel 2 which was used to test the limitations due to overlap of atrial activity and stimulation artifact.*
